# Resolved but not forgotten: Stroop conflict dredges up the past

**DOI:** 10.3389/fpsyg.2014.01327

**Published:** 2014-11-20

**Authors:** Eliot Hazeltine, J. Toby Mordkoff

**Affiliations:** Department of Psychology, University of IowaIowa City, IA, USA

**Keywords:** conflict adaptation, contingency, executive control, response conflict, response conflict adaptation, congruency sequence effect, ISPC effect

## Abstract

The magnitude of congruency effects depends on, among other things, the specifics of previous trials. To explain these modulating effects, a host of mechanisms by which previous trials affect the processing of relevant and irrelevant information on the present trial have been proposed, including feature repetition advantages, negative priming, item-specific proportion congruency (ISPC) effects, display frequency effects, and sequential modulations of both congruency and frequency effects. However, few experiments have been designed to independently manipulate these factors. In the present study, we used a four-choice Stroop task in which we hold constant the frequencies of the stimulus features and responses, but manipulate the frequencies of their conjunctions. We modified the procedure used by Jacoby et al. (2003), under which the possible word–color pairings differed in terms of proportion occurrence, by adding neutral trials to obtain independent estimates of the effects of display frequency. The results indicate that feature repetitions, display frequency, and sequential modulations of both congruency and frequency effects all affect response time. However, no evidence for an ISPC effect was obtained; the display frequency effect measured on the neutral trials accounted for all differences in the congruency effect, as proposed by Schmidt and Besner (2008). Sequential modulations of congruency effects were observed when the overall proportion of congruent trials was held to a chance level and marginal display frequency was also held constant.

## INTRODUCTION

Our perceptual worlds are cluttered with information, only a small fraction of which should drive behavior at a given time. Thus, it is necessary to differentiate between behaviorally relevant and irrelevant sources of information so that we do not reflexively act on the biggest, shiniest object we perceive. To study selection processes, researchers use the Stroop (1935), Simon (1969), and Eriksen and Eriksen (1974) tasks. These have revealed that selection is imperfect; performance is typically worse on incongruent trials, where the irrelevant and relevant sources of information indicate different responses, than on congruent trials, where the irrelevant and relevant sources of information indicate the same response.

### SEQUENTIAL MODULATIONS OF CONGRUENCY EFFECTS

Among the many factors influencing the effectiveness of our ability to select a source of information are immediately previous events. Gratton et al. (1992) is credited as being the first to report that the magnitude of the difference in response times (RTs) between incongruent and congruent trials is larger following a congruent trial than following an incongruent trial. This phenomenon has since been given many names, including conflict adaptation (Botvinick et al., 2001), the Gratton effect (Notebaert and Verguts, 2008), sequential modulation (Hazeltine et al., 2011b), and the congruence sequence effect (Lee and Cho, 2013). Given that one goal of this paper is to examine the various sources that might contribute to this effect, we will use the atheoretical term “sequential modulation.”

When discovered in a flanker task, sequential modulations were thought to reflect the operation of control mechanisms that dynamically weight the various sources on information in concert with task goals. Following Botvinick et al.’s (2001) influential paper proposing a model in which response conflict triggered a control process that changes the relative weightings of task-relevant and task-irrelevant information, a sizeable literature emerged examining sequential modulations (e.g., Ullsperger et al., 2005; Wendt et al., 2006; Akçay and Hazeltine, 2007, 2011; Chen and Melara, 2009; Schmidt and De Houwer, 2011; Schmidt, 2013a), their time course (e.g., Notebaert et al., 2006; Egner et al., 2010; Duthoo et al., 2014), and their boundary conditions (e.g., Kiesel et al., 2006; Egner et al., 2007; Freitas et al., 2007; Akçay and Hazeltine, 2008; Notebaert and Verguts, 2008; Funes et al., 2010; Hazeltine et al., 2011b; Lee and Cho, 2013; Braem et al., 2014; Kim and Cho, 2014).

However, as researchers have probed deeper into this phenomenon, a host of potential ways that a previous trial can affect the current one has emerged (see Schmidt, 2013a). The claim that sequential modulations reflected changes in the weighting of particular stimuli or stimulus dimensions was first challenged by Mayr et al. (2003), who noted that many experiments examining sequential modulations used two-choice tasks so that when a congruent trial followed a congruent trial or an incongruent trial followed an incongruent trial, exact repetitions of the stimuli were possible, but when an incongruent trial followed a congruent trial or a congruent trial followed an incongruent trial, no exact repetitions were possible. In other words, the shorter RTs stemming from exact repetitions of stimuli (and the absence of the requirement to rebind stimulus features, see Hommel et al., 2004) only benefits congruent trials following congruent trials and/or incongruent trials following incongruent trials. Thus, the pattern of RTs attributed to control processes changing the weightings of various sources of information could be accounted for simply in terms of the effects of repetitions and alternations of stimulus features.

To address this confound, many researchers turned to four-choice tasks, in which all types of congruency sequences can be obtained using stimulus features that did not appear on the immediately preceding trial. Many studies (Ullsperger et al., 2005; Akçay and Hazeltine, 2011; Hazeltine et al., 2011a,b; Lee and Cho, 2013; Kim and Cho, 2014) restrict the analyses of sequential effects to complete alternations, which can be done for all two-trial sequences of congruent and incongruent trials when the task is four-choice. It is also possible to remove trials in which the irrelevant feature on the previous trial indicates the same response as the relevant feature on the current trial (negative priming trials) and trials in which the relevant feature of the previous trial indicates the same response as the irrelevant feature of the current trial, given that these types of transitions may also affect RT, but these are less consistently eliminated from analyses of sequential modulations.

However, the use of four-choice tasks, even when all types of repetitions are removed, can give rise to additional issues for examining sequential modulations. In a typical four-choice conflict task, there are four possible relevant stimulus features each associated with a unique response and four possible irrelevant stimulus features each associated with one of those responses. When the relevant and irrelevant features are randomly paired, only 1/4 of the trials are congruent. Thus, a sequence of two congruent trials represents only 1/16 of the two-trial sequences, whereas a sequence of two incongruent trials, for example, represents 9/16 of the two-trial sequences. This imbalance changes depending on which feature repetitions are eliminated from the analyses (for a full discussion of this issue, see Mordkoff, 2012), but some researchers (e.g., Akçay and Hazeltine, 2007, 2011; Hazeltine et al., 2011b) have opted to increase the rate of congruent trials to obtain more balanced numbers of trials in each of the cells for the analysis, as well as to maintain an equal probability of congruent and incongruent trials.

### DISPLAY FREQUENCY AND CONTINGENCY

And yet increasing the probability of congruent trials causes at least two new confounds with other potential contributors to RT: display frequency and contingency. Display frequency refers to the likelihood of a particular stimulus (i.e., combination of relevant and irrelevant information) appearing on a given trial. To make congruent trials as frequent as incongruent trials in standard four-choice designs as described above, each congruent stimulus must appear three times as often as any given incongruent stimulus, because there are three times as many incongruent stimuli as congruent stimuli. It has been shown that more frequently presented stimuli produce shorter RTs than less frequently presented stimuli (Hick, 1952; Hyman, 1953).

Contingency effects emerge when the relative likelihood of a particular task-relevant feature or response given a particular task-irrelevant feature is different from the overall (or unconditional) probability of that particular task-relevant feature or response. For example, in a Stroop task where the red-colored stimuli are the word “RED” on 1/2 of the trials, and the words “GREEN,” “BLUE,” and “YELLOW” each appear 1/6 each, not only is RED-in-red more frequent than GREEN-, BLUE-, or YELLOW-in-red, but contingencies now exist between the task-irrelevant word “RED” and the task-relevant red color and, therefore, the “red” response. Thus, even though participants are instructed not to attend to the word, it contains information indicating the likely response. Humans are highly sensitive to these contingencies, even when they occur in sources that are to be ignored (Miller, 1987; Mordkoff, 1996).

In this way, both display frequency and contingency may act to reduce RTs to congruent stimuli. Note that in Stroop tasks, where there is a one-to-one mapping between values of the relevant feature and the responses, it is not possible to distinguish between display frequency effects and contingency effects. Note, also, that there is evidence that performance can be affected by the frequency and contingencies that are associated with conjunctions of features, even when these conjunctions are unattended (Mordkoff and Halterman, 2008). While the effects of frequency and contingency are typically insufficient on their own to account for sequential modulations, they can contaminate measures of the congruency. Also, there is evidence that contingency effects may themselves be subject to sequential modulations (Schmidt et al., 2007). That is, the effects of contingency may be larger after trials in which a more frequent pairing of irrelevant and relevant information was presented than after trials in which a less frequent pairing was presented. Thus, the sequential modulation of contingency may be misinterpreted as the more standard modulation of congruency if congruency and contingency are confounded.

### DISPLAY FREQUENCY, CONTINGENCY, AND THE ITEM-SPECIFIC PROPORTION CONGRUENCY (ISPC) EFFECT

Display frequency and contingency effects not only complicate the interpretation of sequential modulations, but, as noted by Schmidt and Besner (2008), they can also provide an alternative explanation for the item-specific proportion congruency (ISPC) effect reported by Jacoby et al. (2003). It had been established that the magnitude of congruency effects depended on the overall proportion of congruent trials (Logan and Zbrodoff, 1979; Tzelgov et al., 1992), but Jacoby et al. (2003) found that the magnitude of the congruency effect for a given task-irrelevant feature can depend on the proportion of trials on which that particular feature is paired with a congruent versus incongruent task-relevant feature. Jacoby et al. (2003) concluded that item-specific processes modulate the influence of task-irrelevant information in the Stroop task, a proposal that has been incorporated into recent models of sequential modulations (e.g., Verguts and Notebaert, 2008; Blais and Verguts, 2012). The proposal that control is implemented in a feature-specific manner has broad implications for theories of attention and executive function, so determining whether the ISPC effect does indeed reflect the tracking of the usefulness of individual feature values is a critical issue.

However, in Stroop tasks in which some word–color pairs are presented more frequently than others, irrelevant features become predictive of both relevant features and correct responses, and Schmidt and Besner (2008) showed that such contingencies could completely explain the pattern of results without appealing to differences in attentional control (see also, Grandjean et al., 2013). While both frequency and contingency provide possible explanations for the ISPC effect, the design used by Jacoby et al. (2003) did not allow for the independent measurement of these effects, because both were confounded with the putative ISPC effect. To address this, Schmidt (2013b) designed a Stroop task in which there were three types of incongruent trials: frequently paired colors and words with words that were usually incongruent (high/low), infrequently paired colors and words with words that were usually incongruent (low/low), and infrequently paired colors and words with words that were usually congruent (low/high). The high/low were performed 40 ms faster than the low/low trials, indicating a robust contingency effect, but there was no difference in RT for the low/high and low/low trials, suggesting that the proportion congruence had little impact on performance.

Thus, there is evidence to suggest that the ISPC is really driven by contingency. Here, we directly test whether ISPC effects and display- frequency/contingency effects are approximately the same size to determine if the latter can account for the former. In essence, the question is whether a specific feature value (e.g., the word “red”) can be associated with something abstract like congruence or incongruence rather than being associated with a particular response.

## EXPERIMENT

The goal of the present study was to tease apart the various potential modulators of congruency effects: repetition effects, frequency (and contingency) effects, and sequential modulations. To do this, we use a four-choice Stroop task in which we hold constant the marginal frequencies of the relevant and irrelevant stimulus features, as well as the responses, but manipulate the frequencies of their conjunctions. We modify the procedure used by Jacoby et al. (2003) and Schmidt and Besner (2008) in which the possible word–color pairings differ in terms of the proportion of trials on which they occur (display frequency; see also, Schmidt, 2013b). In a Stroop task where there is a one-to-one mapping between the relevant stimulus feature (color) and the response, changing the display frequencies of individual color/word pairings necessarily changes the proportion of congruence for the particular words. That is, the proportion congruence of a word can be increased only by making the congruent color/word pairs more frequent than the incongruent color/word pairs. In order to decrease the proportion congruence of a word, it must be presented in an incongruent color more frequently than in the congruent color. Therefore, to obtain separate and independent measures of the effects of frequency in the absence of congruency, we include neutral trials. On these trials, the irrelevant word is not associated with an option within the response set, but different words appear more frequently in some colors than in others, matching (exactly) the frequency differences of the congruent and incongruent trials.

### METHOD

#### Participants

One hundred and six undergraduate students (58 females) at the University of Iowa participated to fulfill their course requirements. All were self-reported to be native English speakers with corrected-to-normal vision. Participants provided informed consent but were naïve to the study’s design and purpose.

#### Stimuli and apparatus

The stimuli were presented against a black background on a 17 inch LCD monitor of a personal computer. The viewing distance was approximately 120 cm. Visual basic software was used to control and present the stimuli. The Speech Recognition software from Windows XP was used to record the RT and accuracy of each trial.

Four ink colors (red, yellow, green, and blue) were paired with four color words (RED, BLUE, YELLOW, and GREEN) and four neutral words (CAR, LINE, FOLDER, and SHIRT) to form Stroop trials and neutral trials (see **Table 1**). All colors were presented at chance level (16 of 64 presentations) and all words were presented at chance level (8 of 64 presentations), as well. The overall proportion congruent of the block was at chance level (8 of 64 presentations). The trial frequencies of the neutral trials were selected to match those of the congruent and incongruent Stroop trials. The four neutral words (CAR, LINE, FOLDER, and SHIRT) were chosen to match the length of the color words.

**Table 1:** Example design in terms of display frequency as a function of task-relevant color and task-irrelevant word.

	Word (congruent/incongruent trials)				Word (neutral trials)			
Color	RED	BLUE	YELLOW	GREEN	CAR	LINE	FOLDER	SHIRT
Red	**1**	2	4	1	1	2	4	1
Blue	4	**1**	1	2	4	1	1	2
Yellow	1	4	**2**	1	1	4	2	1
Green	2	1	1	**4**	2	1	1	4
PC	1/2 C	1/2 C	C	2 C				

*Row labels (in lower case) indicate colors; column labels (in upper case) indicate words. Table entries (numbers) indicate trials per block. Numbers in bold indicate congruent trials. PC indicates the proportion congruence for a word; C, chance*.

To manipulate item frequency, the pairings of colors and words was arranged so that for each color there was one color word and one non-color word paired with it four times every 64 trials (frequent pairing), one color word and one non-color word paired with it twice every 64 trials (moderate pairing), and two color words and two non-color words paired with it once every 64 trials (infrequent pairing; see **Table 1**). Participants were randomly assigned to one of four color-word pairing mappings so that the roles of the four colors were counterbalanced across participants. With this arrangement, we were able to vary the frequencies for the contingencies between words and colors while having a neutral match for each congruent and incongruent word.

#### Procedure

Each trial started with a fixation cross of 500 ms. After a blank of 300 ms, target stimulus was presented. Participants were instructed to name the ink color as quickly and accurately as possible. Participants had 5,000 ms to respond. After an incorrect response, a display with the words “you said:” followed by the word recorded by the voice recognition system on one line and “correct response:” followed by the correct color name on another line. All of the words in the error display were white presented on a black background. The incorrect response and correct word were presented in white for 1000 ms following an error trial. After a correct response or the error display, a blank display was presented for 700 ms. Participants performed 15 blocks of 64 trials each. The first two blocks were treated as practice blocks.

### RESULTS AND DISCUSSION

Two participants were removed from the analysis due to the malfunctioning of the speech recognition system. The mean proportion correct was 0.97. Inspection of the cell means of accuracy indicated that any effects of accuracy would be small (<3%), so our analyses focused on RT. The first two trials of each block, error trials (2.8%), trials immediately following an error trial (2.8%), and trials with RTs less than 150 ms (3.0%) or greater than 2,000 ms (0.3%) were excluded from the analysis.

#### Repetition effects

Our questions concern the various ways that the composition of previous trials affects performance on subsequent trials (see Dalrymple-Alford and Budayr, 1966), so we first assessed the effects relating to feature repetitions. Five types of trials were considered: trials with no repeated features (NO), trials in which the relevant feature (color) repeated (CC), trials in which the irrelevant feature (word) repeated (WW), trials in which the relevant feature on the previous trial indicated the same response as the irrelevant feature on the current trial (CW), and trials in which the irrelevant feature on the previous trial indicated the same response as the relevant feature on the current trial (WC). We eliminated from the analysis trials in which multiple forms of repetition occurred. Because the frequencies of these various forms of repetitions differ depending on the congruency of the previous and current trial, we restricted our analyses to incongruent trials that followed incongruent trials. Furthermore, to avoid any confounding effects of frequency (the mean frequencies of the four repetition types and control trials differed), we also restricted the analyses to frequent color-word pairings. It was not possible to hold constant the frequency of the previous trial, because this eliminated the possibility of WW and CC trials. Moreover, with these restrictions, it was not possible to perform an ANOVA with the presence/absence of each type of repetition as a factor. Instead, we performed a one-way ANOVA on these five trial types, which revealed a significant effect, *F*(4,408) = 34.26, *p* < 0.0001.

**FIGURE 1:**
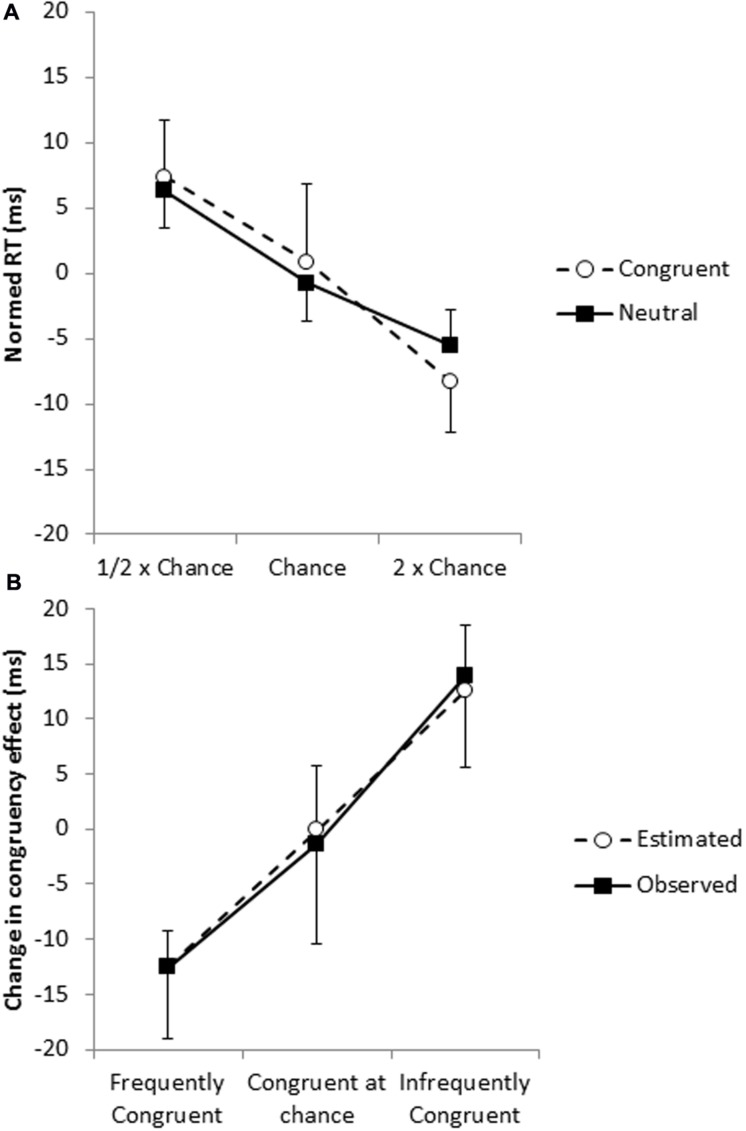
**Mean response times (RTs) for the no repetition trials (None) and trials with just one of the four types of repetitions: Color→Color (CC), Word→Word (WW), Color→Word (CW), and Word→Color (WC).** Asterisks indicate significant differences from the No repetition condition, *p < 0.05; **p < 0.0001.

To examine this finding more closely, we directly compared each repetition type (CC, CW, WC, and WW) to the NO trials (**Figure 1**). Because our focus was on the potential contaminating effect of these repetitions on measures of congruency rather than on the repetition effects themselves, we adopted a liberal statistical threshold uncorrected for multiple comparisons. RTs on the trials on which only color repeated (CC) were 109 ms shorter (575 ms) than RTs on NO trials, *t*(103) = 8.16; *p* < 0.0001. RTs on the trials on which only the word repeated (WW) were 31 ms shorter (653 ms) than RTs on NO trials, *t*(103) = 2.40; *p*< 0.05. Thus, the benefit associated with having to inhibit the same irrelevant word that had to be inhibited on the previous trial was larger than any cost associated with rebinding a repeated word with a novel color. The mean RT for CW trials (657 ms) was 27 ms faster than for NO trials, *t*(103) = 2.42; *p*< 0.05. Because we analyzed only incongruent trials, this result suggests that it is easier to suppress an inappropriate response when it was produced on the immediately preceding trial. Finally, the mean RT on WC trials (690 ms) was 6 ms longer than NO trials, but this difference was not significant, *t*< 1. Thus, there was little evidence that this form of negative priming affected RT in this experiment.

The absence of any costs associated with partial repetitions was unexpected (see, e.g., Hommel, 1998), so to further examine whether rebinding costs played a role in RT, we examined sequences of neutral trials in which the irrelevant word was not associated with a color and therefore should not have produced much response competition. Frequent neutral word–color combinations following neutral word–color combinations produced mean RTs of 578 ms when no features repeated. When only the word repeated, these trials produced RTs of 582 ms, which was not significantly different, *t*< 1. This finding suggests that rebinding costs did not play a major role in the RT in the present experiment, possibly because there were many possible relevant and irrelevant features.

#### Frequency and the ISPC effect

Three of the four types of repetition (CC, WW, CW) produced effects on RT on their own that were similar or greater in magnitude to some modulations of congruency effects. Therefore, we adopted a conservative approach and eliminated trials with any of the four possible forms of repetitions before testing whether display frequency is sufficient to account for the ISPC effect. Moreover, because frequency may modulate congruency effects, as in the ISPC effect, we first examined frequency in the neutral trials only. Note that with the present design, the effects of display frequency and contingency are confounded, so we use the term “frequency” to refer to the combined effects of both. An estimate of the frequency effect was obtained with a one-way ANOVA, which revealed a significant effect, *F*(2,206) = 10.02, *p*< 0.0001, MSE = 356.16, $\eta_{p}^{2}$ = 0.09. Within the neutral trials, frequent combinations of relevant and irrelevant features produced RTs of 577 ms, chance combinations produced RTs of 582 ms, and infrequent combinations produced RTs of 589 ms.

Our next step was to determine whether the frequency effect could account for any observed ISPC effect in the congruent and incongruent trials. Thus, we first determined whether the data indicated ISPC effects and then assessed whether this effect could be explained by frequency as measured in the neutral trials. Therefore, we categorized each trial according to whether the task-irrelevant word was paired with a congruent color frequently (1/2 of trials that the word appeared), at chance (1/4 of trials) or infrequently (1/8 of trials; see **Table 1**) as in Schmidt and Besner (2008). The data were then submitted to a two-way ANOVA with this factor and congruency (without the neutral trials).

There was a significant main effect for congruency, *F*(1,103) = 241.34, *p*< 0.0001, MSE = 6158.87, *$\eta_{p}^{2}$* = 0.70, but not for the proportion of congruency of the word, *F*< 1. Critically, the interaction between the two factors was significant, *F*(2,206) = 4.59, *p*< 0.05, MSE = 1972.60, *$\eta_{p}^{2}$* = 0.43, indicating a significant ISPC effect. When the irrelevant word was frequently congruent, the congruency effect was 111 ms (incongruent 688 ms; congruent 577 ms). When the irrelevant word was congruent at a chance rate, the congruency effect was 97 ms (incongruent 683 ms; congruent 586 ms), and when the irrelevant word was infrequently congruent, the congruency effect was 85 ms (incongruent 678 ms; congruent 593 ms).

**FIGURE 2:**
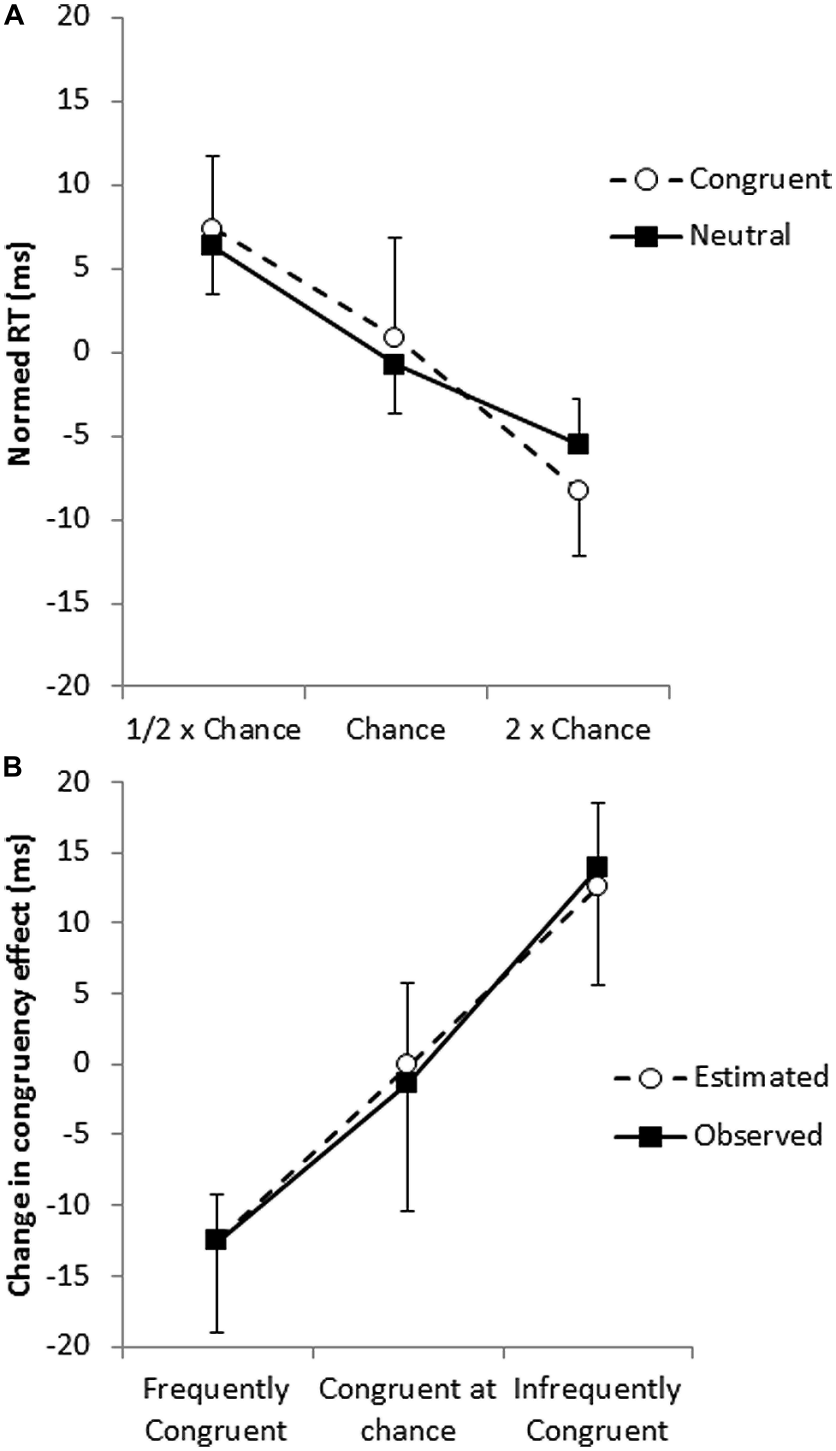
**(A)** Frequency effects for congruent (open circles) and neutral (filled squares) trials with the overall mean of the trial type (congruent or neutral) subtracted out. **(B)** The change in the magnitude of the congruency effect (the ISPC effect) as observed in the congruent and incongruent trials (filled squares) and estimated from the neutral trials (open circles).

However, as pointed out by Schmidt and Besner (2008), this analysis confounds frequency and ISPC effects, so we next examined whether this effect could be accounted for with the frequency effect as measured in the neutral trials. For congruent trials, pairs that include frequently congruent words are themselves more frequent; that is, the frequency of the word–color combination and the proportion that the word is paired with a congruent color is perfectly confounded for congruent trials. Thus, differences in the effect of frequency on the congruent trials and the effect of frequency on the neutral trials provide evidence for an ISPC effect. Frequent congruent trials were performed 16 ms faster than infrequent congruent trials (577 vs. 593 ms), and frequent neutral trials were performed 12 ms faster than infrequent neutrals trials (577 vs. 589 ms); the magnitude of the frequency effect did not differ for the two trial types, *t*< 1 (**Figure 2A**), so it does not appear that the RTs of congruent trials are affected by the proportion of trials in which the word is congruent beyond what would be expected by the proportion of trials in which the word is paired with that color.

Of course, congruent trials represent only one half of the congruency effect, and thus do not provide a strong test of whether the ISPC effect can be accounted for by frequency effects on their own. However, while frequency and proportion congruent are perfectly confounded in the congruent trials, the relationship between the two factors is much more complex for the incongruent trials. For example, the infrequent incongruent stimuli are composed of words that are frequently incongruent, incongruent at chance rate and infrequently incongruent. In short, the effect of frequency on the incongruent trials is less informative regarding the effect of proportion congruency than the congruent trials, because of the complex relationship between the two factors.

The critical question is whether the observed frequency effect in the neutrals can account for the interaction between frequency and proportion congruent (that is, the ISPC effect) in the congruent and incongruent trials. However, when determining the congruency effect for frequently congruent words, for example, RTs from trials in which the word is frequently paired with the congruent color are compared to RTs from trials in which the same word is paired with other colors that it appears with less frequently. Similarly, when determining the congruency effect for the infrequently congruent words, RTs from trials in which the word is infrequently paired with the congruent color are compared to RTs from trials in which the same word is paired with a mixture of other colors that were not infrequently paired with the word.

Therefore, to evaluate whether the ISPC effect could be explained by display frequency, we calculated for each participant the expected change in the magnitude of the congruency effect based on the frequency effect observed in the neutral trials and the proportions of frequent, chance, and infrequent pairings making up the incongruent trials. This procedure predicted a 25 ms change in the congruency effect between trials with frequently congruently words and trials with infrequently congruent words, which was similar to the observed 26 ms change in the congruency effect (i.e., the ISPC effect), *t*< 1 (**Figure 2B**). In short, it appears that display frequency can account for the ISPC effect without assuming that individual irrelevant items have individually modulated congruency effects based on the likelihood that the irrelevant item is congruent. Consistent with the conclusions of Schmidt and Besner (2008), there is no evidence for item-specific control processes.

#### Sequential modulations

We now return to the debate about whether sequential modulations of congruency effects can be observed without repetition and frequency confounds (e.g., Mayr et al., 2003; Hommel et al., 2004; Ullsperger et al., 2005; Akçay and Hazeltine, 2007, 2011; Hazeltine et al., 2011b; Schmidt, 2013a). To examine this in the present data set, we selected the trials without any of the four types of repetitions and looked only at the most frequent combinations of colors and words because, with repetitions removed, the different types of transitions consist of trials with different mean frequencies. These criteria led to five participants not having any trials in which a congruent trial followed a congruent trial, so these individuals were removed from the analysis. The data from remaining 99 participants were submitted to a two-way ANOVA with previous congruency and current congruency as factors. This produced main effects previous congruency, *F*(2,196) = 15.86, *p*< 0.0001, MSE = 3833.75, *$\eta_{p}^{2}$* = 0.14, congruency, *F*(2,196) = 169.41, *p*< 0.0001, MSE = 6259.28, *$\eta_{p}^{2}$* = 0.63, and an interaction between the two factors, *F*(4,392) = 6.45, *p*< 0.0001, MSE = 2706.71, *$\eta_{p}^{2}$* = 0.06.

**FIGURE 3:**
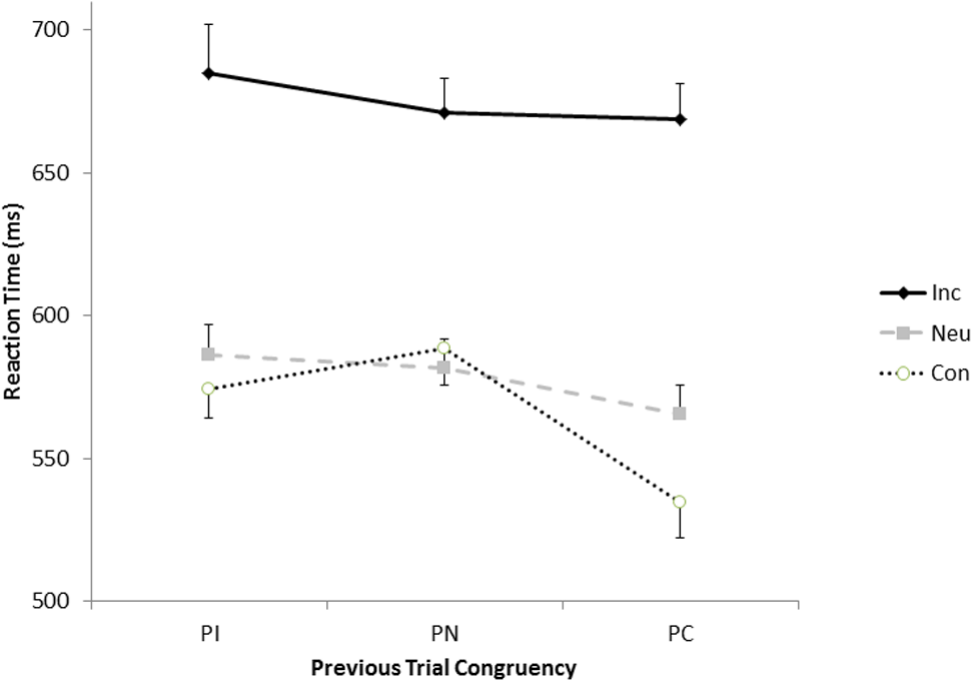
**Response times for the congruent (open circles), neutral (gray squares), and incongruent (black diamonds) as a function of the congruency of the previous trials**.

As depicted in **Figure 3**, the congruency of the previous trial affects the magnitude of the congruency on the current trial, even when repetitions and contingency effects are accounted for. The pattern is somewhat atypical in that the smallest congruency effects are observed after neutral trials (83 ms) rather than after incongruent trials (111 ms). As is typical when sequential modulations are observed, the congruency effect was largest (134 ms) after congruent trials.

Finally, we examined whether the frequency effect was moderated by the frequency of the previous trial (see, Schmidt et al., 2007). To do this, we looked at neutral trials that followed neutral trials, so that sequential modulations of frequency effects would not be confounded with sequential modulations of congruency effects. A two-way ANOVA with current frequency and previous frequency as factors, looking only at trials with no repetitions of any kind, revealed a significant effect of current frequency, *F*(2,206) = 3.77, *p*< 0.05, MSE = 2271.17, *$\eta_{p}^{2}$* = 0.04, and a significant interaction between current frequency × previous frequency, *F*(4,412) = 2.41, *p*< 0.05, MSE = 2364.36, *$\eta_{p}^{2}$* = 0.02. The difference between frequent items and infrequent items was 1 ms following infrequent items, 21 ms following moderately frequent items and 7 ms following frequent items. Thus, while the interaction was significant, replicating Schmidt et al. (2007), the effect appeared to be small and, as above, non-monotonic. Nonetheless, this finding indicates that when congruency is confounded with frequency, the source of sequential modulations is ambiguous.

## GENERAL DISCUSSION

In the present study we sought to assess the various contributors to the magnitude of congruency effects using a four-choice Stroop task with neutral trials and a large number of participants. The results indicate that the majority factors did indeed impact performance, making them candidate sources of changes in the congruency effect. Feature repetitions, frequency, and sequential modulations of both congruency and frequency all produced significant effects on RT when the other factors were held constant.

The present findings make three principle contributions. First and most generally, they indicate RTs depend on a host of factors. While these factors are difficult to dissociate, in the present study we ran a relatively large number of subjects and manipulated item frequency independently from the frequency of individual features (e.g., the color blue or the word “shirt”), which was held constant. Under these conditions, it was apparent that repetitions of irrelevant sources of information shortened RT.

Second, the findings demonstrate that trial-frequency effects (and/or contingency effects), as measured in neutral trials, are of a magnitude that is sufficient to allow them to account for the ISPC effect on their own. That is, while previous work (e.g., Schmidt and Besner, 2008; Schmidt, 2013b) demonstrated that contingency effects provided a possible explanation for the ISPC effect, the present findings indicate that the ISPC effect is the same magnitude as what is predicted by the contingency effect, as measured in the neutral trials. Thus, we conclude that there is no evidence for an ISPC effect.

Third, the findings indicate that sequential modulation of congruency effects can be observed when feature repetitions, negative priming, and frequency effects are all controlled, and congruent and incongruent trials occur at overall frequencies equal to chance. While previous studies have shown that sequential modulations can occur without contingency effects (e.g., Kim and Cho, 2014; Schmidt and Weissman, 2014; Weissman et al., 2014), the present study shows that sequential modulations and contingency effects can co-occur, indicating that the former do not only emerge in the absence of the latter (see Bugg, 2014). Moreover, just one eighth of the trials in the present experiment were congruent, which may account for the somewhat unusual pattern of sequential modulations (**Figure 3**). The relative relatedness between the congruent and incongruent words may also have affected the pattern of sequential modulations, just as it may have affected the pattern of partial repetitions. This would explain why congruency effects were smallest after neutral trials rather than after incongruent trials. In any case, the data suggest that the congruency of the previous trial can affect the magnitude of the congruency effect on the current trial independent of feature repetitions and display frequency.

In sum, the data indicate that RTs reflect a set of processes that are sensitive to a range of factors that include both specific (e.g., the individual features) and abstract (e.g., the congruency and frequency of the conjoined item) information relating to the previous trial. However, there is no evidence that modulations of the congruency effect are implemented at the level of individual features; that is, there is no evidence for an ISPC. On the other hand, sequential modulations of congruency effects are apparent when repetitions are eliminated and display frequencies held constant. However, given that the events of the previous trial affect the processing of the current trial in a myriad of ways, sequential modulations may be difficult to study using tasks that do not allow for the various contributors to be isolated and estimated. As in other domains, the resolution of conflict is complicated when so little of the past is forgotten.

## Conflict of Interest Statement

The authors declare that the research was conducted in the absence of any commercial or financial relationships that could be construed as a potential conflict of interest.
